# Hospital Outcomes in Antepartum Mental Disorders: A Study on 897,397 Pregnant Inpatients

**DOI:** 10.3390/bs9100105

**Published:** 2019-09-29

**Authors:** Narmada N. Bhimanadham, Pranita Mainali, Chris A. Robert, Anum Masroor, Henry K. Onyeaka, Sadaf Hossain, Rikinkumar S. Patel

**Affiliations:** 1 Department of Public Administration, Drake University, Des Moines, IA 50311, USA; drneerjabn@yahoo.com; 2 Department of Psychiatry, Washington DC VA Medical Center, Washington, DC 20422, USA; pranita_mainali@icloud.com; 3 Department of Gynecology, Rao Hospital, Coimbatore 641002, India; drchrisandrea@gmail.com; 4 Department of Psychiatry, Khyber Medical College, Khyber Pakhtunkhwa 25120, Pakistan; Anum180@hotmail.com; 5 Department of Public Health, Harvard School of Public Health, Boston, MA 02115, USA; henryonyeaka@hsph.harvard.edu; 6 Department of Psychiatry, Brookdale Hospital and Medical Center, Brooklyn, NY 11212, USA; hossainsadaf073@gmail.com; 7 Department of Psychiatry, Griffin Memorial Hospital, Norman, OK 73071, USA

**Keywords:** pregnancy, complications, antepartum, mental illness, depression, outcomes, psychiatric disorder

## Abstract

Objective: To evaluate the impact of antepartum mental disorders (AMD) in medical and psychiatric comorbidities, and inpatient outcomes during hospitalizations for pregnancy/birth-related complications. Methods: We used the national inpatient sample (NIS) data and included 19,170,562 female patients (age, 12–40 years) with a principal diagnosis of pregnancy/birth-related complications and grouped by co-diagnoses of AMD (N = 897,397). We used a binomial logistic regression model to evaluate the odds ratio (OR) for major severity of illness and adjusted for demographic confounders. Results: The hospitalizations with AMD increased by 22.1% (*p* < 0.001) from 2010 to 2014. White females (66.1%) and those from low-income families (<25th percentile, 31.8%) majorly had comorbid AMD. Depression (43.8%) and drug abuse (27%) were prevalent psychiatric disorders in AMD inpatients. Comorbid AMD inpatients had a higher likelihood for major severity of illness (OR 2.475, 95% CI 2.459–2.491, *p* < 0.001). They also had a longer hospitalization stay with a mean difference of 0.486 days (95% CI 0.480–0.491) and higher total charges by $1889.420 per admission (95% CI 1852.670–1926.170) than non-AMD inpatients. Conclusions: AMD is associated with worsening of severity of illness in pregnancy/birth-related complications and require acute inpatient care. Mental health assessment and treatment of AMD, and education about efficacy and safety of psychiatric medications may help to improve outcomes in these patients.

## 1. Introduction

As per the World Health Organization, 10% of pregnant women and 13% who have recently delivered have experienced mental disorders worldwide, with an upward trend in developing countries [1]. The effect of mental disorders in the antepartum and postpartum period have numerous unwanted effects such as impact on the developing fetus resulting in preterm birth and low birth weight [2], and affects the cognitive behavior of the children [3]. These women with antepartum mental disorders at lower maternal age and per capita income have a strong correlation with psychiatric illnesses five to eight years after delivery [4].

Mental disorders in the antenatal period are associated with a variety of complications related to pregnancy and delivery. These complications are directly related to the severity of mental health conditions. Depression and anxiety during the antenatal period have shown strong association with increased nausea and vomiting, prolonged sick leaves during pregnancy, and frequent obstetrician visits specifically related to fear of childbirth and contractions [5]. Depression in the later part of pregnancy is associated with an increased risk of epidural analgesia, operative deliveries, and admissions in the neonatal care unit [6] that may affect the psychological wellbeing of both mother and child. Anemia and polyhydramnios are highly prevalent in patients with panic disorder [7]. Schizophrenia and affective disorder patients, on the other hand, are more likely associated with placental abnormality leading to placental abruptio, and antenatal hemorrhage causing fetal distress [8]. As per the recent data from Substance Abuse and Mental Health Services Administration (SAMHSA), the percentage of pregnant women taking alcohol with or without drugs has declined from 46.6% to 34.8% from 2000 to 2010, though it has increased from 51.1% to 63.8% in the same group for drug abuse [9].

So, we conducted a nationwide study of hospitalizations for pregnancy or birth-related complications and evaluated the differences in demographic characteristics, medical and psychiatric comorbidities, and hospital outcomes (severity of illness, length of stay (LOS), and total charges) between inpatients with versus without co-diagnosis of antepartum mental disorders (AMD), i.e., mental disorders during the perinatal period/pregnancy.

## 2. Methods

### 2.1. Data Source

A retrospective cohort study was conducted using the Healthcare Cost and Utilization Project’s (HCUP) national inpatient sample (NIS) data from January 2010 to December 2014 [10]. The NIS database is most commonly utilized to evaluate patterns in demographics and hospital outcomes. It is the largest inpatient data that covers 4411 hospitals across 45 states in the United States [10]. To protect the privacy of patients, physicians, and hospitals, the identifiers were de-identified using KEY_ID during HCUP NIS data processing [11]. Thus, we did not require the Institutional Review Board’s permission to conduct a study on publicly available de-identified inpatient data.

### 2.2. Inclusion and Exclusion Criteria

We included female patients (age, 12 to 40 years) with a principal diagnosis of pregnancy or birth-related complications based on the clinical classification software (CCS) codes [12] present in the NIS data. The study population (N = 19,170,562) was further grouped by co-diagnoses of mental disorders in pregnancy antepartum (N = 897,397). The international classification of diseases, ninth revision (ICD-9) diagnoses codes used to identify AMD are 648.41 or 648.43.

Female patients below 12 and above 40 years were excluded from our study. We excluded the hospital admission for causes other than pregnancy or birth-related complications.

### 2.3. Variables of Interest

Demographic variables evaluated in this study were age, race, and median household income [11]. To measure the differences in hospital outcomes in inpatients by co-diagnoses of antepartum mental disorders, the following variables were included: Severity of illness, number of chronic conditions, LOS, and total charges [11]. In the NIS, severity of illness means loss of function and was assessed using All Patient Refined Diagnosis Related Groups (APR-DRGs) and 3M-Health Information Systems software. LOS means the number of nights the patient remained in the hospital for the principal diagnosis, and total charges during hospitalization do not include professional fees and noncovered charges [11].

Comorbidities are considered coexisting conditions, and using ICD-9 and CCS codes alcohol abuse/dependence, drug abuse/dependence, depression, psychoses, deficiency anemias, diabetes, hypertension, hypothyroidism, and obesity were identified in the discharge diagnoses from diagnoses fields from DX2 to DX25 in the NIS [11,12].

### 2.4. Statistical Analyses

We used descriptive statistics and Pearson’s chi-square test for categorical data and independent sample *t*-test for the continuous data (including LOS and total charges) to measure the differences in demographic characteristics and hospital outcomes. We applied the discharge weight (DISCWT), which is given in the NIS [11], to attain national representation of the inpatient population. Differences in comorbidities were quantified using Pearson’s chi-square tests. We used a binomial logistic regression model to evaluate the odds ratio (OR) for major severity of illness, with AMD categorical data (yes vs. no as reference category) and adjusted for demographics characteristics (age, race, and median household income). A *p* value < 0.01 was used to determine the statistical significance of the analyses conducted on statistical package for the social sciences (SPSS) version 25 (IBM Corp., Armonk, New York, NY, USA).

## 3. Results

**Figure 1:**
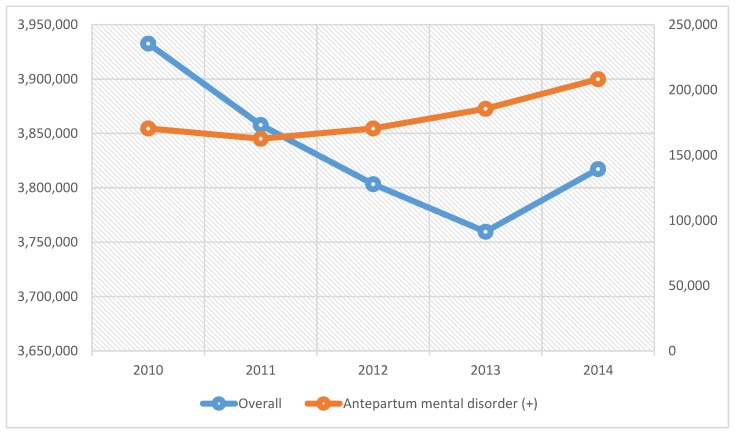
Trends of hospitalizations for pregnancy or birth-related complications and antepartum mental disorders, 2010 to 2014.

We analyzed 19,170,561 hospitalizations for principal discharge diagnoses of pregnancy or birth-related complications of which 897,397 patients (4.68%) had codiagnoses of mental health disorders in pregnancy antepartum or AMD. There was a linear rising trend (increased by 22.1%, *p* < 0.001) of hospitalizations for pregnancy or birth-related complications with AMD from 4.3% (N = 170,509 in 2010) to 5.5% (N = 208,245 in 2014), whereas total number of hospitalizations for pregnancy/birth-related complications decreased by 3% (N = 3,932,616 in 2010 to N = 3,817,160 in 2014) as shown in Figure 1.

### 3.1. Demographic Characteristics

A higher proportion of inpatients with pregnancy/birth-related complications was seen in those 21 to 30 years of age (54.6% with AMD, 53.7% without AMD). White females were majorly affected by AMD (66.1%) during hospitalization compared to other race/ethnicities. About one-third of females from low-income families (below the 25th percentile) had comorbid AMD, and the prevalence decreased with an increase in median household income.

Among medical comorbidities, deficiency anemias were most prevalent in inpatients with AMD (13.9%) and non-AMD (8.9%). Depression was a prevalent psychiatric disorder in AMD inpatients (43.8%) followed by drug abuse (27.3%). The differences in AMD and non-AMD inpatients are shown in Table 1.

### 3.2. Hospitalization Outcomes

As per the adjusted binomial logistic regression model shown in Table 2, 15.4% of AMD inpatients had higher odds for major severity of illness (OR 2.475, 95% CI 2.459 to 2.491, *p* < 0.001) compared to 6.9% non-AMD inpatients.

Pregnant inpatients had a more extended hospitalization stay with a mean difference of 0.486 days (95% CI 0.480 to 0.491, *p* < 0.001) and higher total charges by USD 1889.420 per admission (95% CI 1852.670 to 1926.170, *p* < 0.001) than non-AMD inpatients for pregnancy or birth-related complications management (Table 3).

## 4. Discussion

In our study, we analyzed the data of hospitalized females (age, 12–40 years) with principal discharge diagnoses of pregnancy or birth-related complications and compared the female inpatients with versus without AMD. We found that the hospitalization for pregnancy or birth-related complications with AMD increased by 22% over the five-year study period. These inpatients had 2.5 times higher odds for major severity of illness during hospitalization with longer LOS by 0.5 days and total charges by USD 1889 per inpatient admission compared to pregnant inpatients without AMD.

Pregnancy is a stressful situation where the body goes through several changes, and so it brings its own set of anxiety with it [13]. One in ten women with no previous mental health conditions are subjected to suffer from anxiety and depression in the antenatal period, and the threshold level for the same is far lower in patients suffering from mental health conditions before pregnancy [13]. Many women during pregnancy decide to stop their psychotropic medications with the fear of side effects on the developing fetus [14]. Women suffering from psychiatric conditions have weak social and emotional support and financial issues, and so these factors collectively make psychiatric symptoms worse [15]. As per a study, women who discontinued psychiatric treatment were five times more likely to relapse during pregnancy in comparison to women who continued treatment [14]. Mental health conditions are taboos in some cultures, and women from such cultures are likely to be at higher risk of developing AMD due to the negligence of wellbeing of mental health [16,17]. Some patients prefer not to report or underreport depressive symptoms during pregnancy with the fear of social judgment or losing custody of their children, resulting in severe mental health conditions during pregnancy [18].

Substance abuse is another threat to pregnancy and its adverse outcome, including fetal complications. As per a study, about one-third of patients enrolled in substance abuse programs are female, and 90% of them were in childbearing age (15 years to 39 years) [19]. Women with substance use disorder (SUD) use contraception less often than women without SUD, resulting in unwanted pregnancy and complications associated with substance abuse [20]. This includes physical, intellectual, behavioral, and learning disabilities in a newborn with alcohol abuse, stillbirth and growth retardation with cannabis abuse, and placental abnormalities, stillbirth, and preterm birth with opioid abuse [20]. Accidental pregnancy in women who are actively using illegal drugs puts them at risk of detention, arrest, and punishment resulting in avoiding medical and prenatal care all together [21]. Such patients come to the attention of medical personnel at the time of delivery or to take treatment for the complications associated with their risky behavior [21].

Duration of hospitalization and healthcare expenditures are also high among women with AMD [22]. Women with depression in the antenatal period are at risk of developing postpartum depression, requiring an extended period of hospital stays [23,24]. A recent study states that a third of depression starts in pregnancy or pre-pregnancy, and its chances increase up to three times in the first five weeks of delivery [19]. Women with schizophrenia are at a 1.4-fold increased risk of induction of labor, and emergency and elective cesarean section [25]. Also, neonates born to schizophrenic mothers are at 1.6 times increased risk of premature birth and low Apgar score at 1 min (under 7), and 2.5 times increased risk of resuscitation when compared to neonates born to mothers without schizophrenia [25]. So, all these factors, individually and collectively, increase the LOS and health care cost as seen in our study population in pregnant inpatients with AMD.

Extensive mental health assessment needs to be practiced to prevent AMD and its effect on pregnancy. Mental health assessment should be made a regular practice during general health appointments to catch symptoms in the early phase. Also, women should be assessed during every prenatal appointment for mental health screening [19]. Women need to be educated about the safety and efficacy of psychiatric medications. We also recommend increasing the awareness of mental illnesses by organizing mental health camps, so that the women will not feel ashamed or pass through societal judgment, and fearlessly can accept the mental health condition and seek psychiatric care [19]. Women diagnosed with substance use disorder should be assessed for coexisting psychiatric conditions, and those who are in the reproductive age group should be educated about injectable/long-term contraceptive options [19].

Since the NIS is an administrative database, there exists a lack of patient-level data. Also, re-hospitalizations add to the total inpatient burden and is not accounted for in our study due to the nature of the database. One of the strengths of this study is that the data were collected from the NIS, which is an excellent population-based representation of diseases associated with systematic and temporal factors. This study is the national representation of the population provided by the dataset, as well as a uniform collection of data obtained over five years through ICD-9 codes, adding to the strength of the study. Another strength of this study is its large sample size and the reliability of the data. Given that the information is coded independently by the individual practitioners, therefore, it was subjected to minimal reporting bias.

## 5. Conclusions

A higher proportion of pregnant females with AMD had medical and psychiatric comorbidities that worsened the severity of illness (by 2.5 times) in hospitalizations for pregnancy or birth-related complications. So, these patients required acute inpatient care leading to a more extended hospitalization period and higher total cost compared to the patients without AMD. Mental health assessment and treatment of AMD and education about efficacy and safety of psychiatric medications, including an emphasis on long-term contraception and substance abuse programs, may improve inpatient outcomes and health-related quality of life.

## Figures and Tables

**Table 1:** Demographic predictors and comorbidities in pregnant inpatients with antepartum mental disorders.

Variable	Antepartum Mental Disorders		*p* Value
	No (%)	Yes (%)	
Total Inpatients	18,273,165	897,397	-
Age, in years			
12–20	11.9	12.0	<0.001
21–30	53.7	54.6	
31–40	34.4	33.4	
Race			
White	51.9	66.1	<0.001
Black	15.4	17.1	
Hispanic	21.6	11.4	
Other	11.0	5.4	
Median household income, in percentiles			
0–25th	28.0	31.8	<0.001
26th–50th	25.0	26.6	
51st–75th	25.1	23.9	
76th–100th	21.9	17.7	
Severity of illness, in loss of functions			
Minor	61.5	29.3	<0.001
Moderate	31.6	55.3	
Major	6.9	15.4	
Psychiatric comorbidities			
Depression	0.2	43.8	<0.001
Psychosis	0.1	16.8	<0.001
Alcohol abuse	0.0	2.3	<0.001
Drug abuse	0.4	27.3	<0.001
Medical comorbidities			
Deficiency anemias	8.9	13.9	<0.001
Diabetes	1.1	2.4	0.008
Hypertension	2.3	5.1	0.389
Hypothyroidism	2.5	4.5	<0.001
Obesity	5.6	11.6	<0.001

**Table 2:** Odds of major severity of illness due to antepartum mental disorders.

Variable	Odds Ratio	95% Confidence Interval		*p* Value
		Upper	Lower	
Age, in years				
12–20	Reference			
21–30	0.990	0.984	0.996	0.001
31–40	1.245	1.237	1.252	<0.001
Race				
White	Reference			
Black	1.892	1.883	1.901	<0.001
Hispanic	1.103	1.098	1.109	<0.001
Other	1.079	1.072	1.086	<0.001
Median household income, in percentiles				
0–25th	Reference			
26th–50th	0.930	0.925	0.934	<0.001
51st–75th	0.910	0.906	0.915	<0.001
76th–100th	0.846	0.841	0.850	<0.001
Major severity of illness				
No	Reference			
Yes	2.475	2.459	2.491	<0.001

**Table 3:** Difference in length of stay and charges due to antepartum mental disorders.

Variable	Antepartum Mental Disorders		*t*-Test for Equality of Means		
	No	Yes	Mean Difference	95% Confidence Interval	*p* Value
Mean length of stay (SD), in days	2.67 (2.48)	3.16 (3.78)	0.486	0.480 to 0.491	<0.001
Mean total charges (SD), in USD	15,387 (16,859)	17,276 (20,662)	1889.420	1852.670 to 1926.170	<0.001
